# A-to-I Editing Is Subtype-Specific in Non-Hodgkin Lymphomas

**DOI:** 10.3390/genes15070864

**Published:** 2024-07-01

**Authors:** Cai Chen, Ralf Bundschuh

**Affiliations:** 1 Biophysics Graduate Program, The Ohio State University, Columbus, OH 43210, USA; 2 Center for RNA Biology, The Ohio State University, Columbus, OH 43210, USA; 3 Department of Physics, The Ohio State University, Columbus, OH 43210, USA; 4 Department of Chemistry & Biochemistry, The Ohio State University, Columbus, OH 43210, USA; 5 Division of Hematology, The Ohio State University, Columbus, OH 43210, USA

**Keywords:** RNA editing, RNA-Seq, ADAR, non-Hodgkin lymphomas, gene expression

## Abstract

Cancer is a complex and heterogeneous disease, in which a number of genetic and epigenetic changes occur in tumor onset and progression. Recent studies indicate that changes at the RNA level are also involved in tumorigenesis, such as adenosine-to-inosine (A-to-I) RNA editing. Here, we systematically investigate transcriptome-wide A-to-I editing events in a large number of samples from Non-Hodgkin lymphomas (NHLs). Using a computational pipeline that determines significant differences in editing level between NHL and normal samples at known A-to-I editing sites, we identify a number of differentially edited editing sites between NHL subtypes and normal samples. Most of the differentially edited sites are located in non-coding regions, and many such sites show a strong correlation between gene expression level and editing efficiency, indicating that RNA editing might have direct consequences for the cancer cell’s aberrant gene regulation status in these cases. Moreover, we establish a strong link between RNA editing and NHL by demonstrating that NHL and normal samples and even NHL subtypes can be distinguished based on genome-wide RNA editing profiles alone. Our study establishes a strong link between RNA editing, cancer and aberrant gene regulation in NHL.

## 1. Introduction

Tumor onset and progression involve a number of epigenetic changes, such as dysregulation of DNA methylation and histone modifications. In addition to these well-established mechanisms which occur at the genomic level, post-transcriptional events could affect cell growth and proliferation as well. One such post-transcriptional event is RNA editing, which alters transcribed RNAs, resulting in RNA products different from the genomically encoded sequence. Alteration can occur through the insertion or deletion of nucleotides relative to the original template (insertional/deletional RNA editing), or via substitution, in which one nucleotide is changed to another. RNA editing is widely spread across species and occurs, e.g., in viruses, protozoa, plants, mammals, slime molds and archaea [1,2]. It is absolutely essential for survival in some cases, while it provides another layer of fine tuning of the genetic program for others.

The most common editing type in mammals involves the conversion of adenosine to inosine (A-to-I editing), which is mediated by adenosine deaminase acting on RNA (ADAR) enzymes [3]. ADARs catalyze the hydrolytic deamination reaction within double-stranded RNA (dsRNA) substrates [3]. Inosine preferentially base pairs with cytidine, and is therefore functionally equivalent to guanosine. Thus, A-to-I editing in mRNA can alter the genetic information stored in the primary sequence, leading to changes in protein-coding sequences, mRNA stability and splicing [4,5].

Bioinformatics and high-throughput sequencing studies have identified a large number of such events occurring in both coding and non-coding regions of the human transcriptome [5,6,7,8,9,10,11,12]. While both coding and non-coding sequences undergo A-to-I editing, it has been found that editing occurs mainly in repetitive sequences which are located within introns, 5′ or 3′ untranslated regions (UTRs).

Editing efficiency or editing level for a single editing site represents the proportion in which the edited version (an inosine) occurs at this site, replacing the genomically encoded adenosine, among all RNA molecules of a particular gene. Editing efficiency varies greatly among different adenosines (ranging from almost no editing to 100%) and between different cell types; therefore, different transcript variants from the same genomic sequence are generated, dramatically increasing transcriptome diversity.

It has been shown that for a given site within a certain target and cell type, the variability among different healthy individuals in editing efficiency is low, suggesting editing levels are tightly regulated in vivo [13]. Abnormal A-to-I editing has been linked to a number of diseases, such as dyschromatosis symmetrica hereditaria, amyotrophic lateral sclerosis, epilepsy, depression and schizophrenia [14,15]. A-to-I RNA editing is also severely dysregulated in tumor tissues [16] and ADAR editing activity is related to cell migration and proliferation [17], suggesting it may serve as a potential epigenetic mechanism participating in carcinogenesis. Several studies have reported individual cancer-related A-to-I editing targets, such as increased editing of antizyme inhibitor 1 (AZIN1) in hepatocellular carcinoma (HCC) [18], non-small-cell lung cancers [19], esophageal squamous cell carcinoma [20], and colorectal cancer [21,22,23], decreased editing of glioma-associated oncogene 1 (GLI1) in the hedgehog signaling pathway in basal cell carcinoma tumors [24], decreased editing of Insulin-like growth factor-binding protein-7 (IGFBP7) in non-melanoma skin cancers [25], increased editing of GABRA3 in breast cancer [26] and increased editing of ras homologue family member Q (RHOQ) [27].

Taking advantage of whole-genome and transcriptome deep-sequencing technologies, genome-wide A-to-I editing events in different cell types and healthy tissues have been extensively investigated using bioinformatics tools that are capable of identifying mismatches between RNA reads and the matching reference genome [28,29,30,31] (for a review see [32]). It is now obvious that combining high-throughput sequencing and bioinformatics has the ability to identify RNA editing events that occur at the single-nucleotide level across the whole transcriptome. These results have been collected in several databases, such as, e.g., the Database of RNA editing (DARNED) [33,34], Rigorously Annotated Database of A-to-I RNA editing (RADAR) [35] and REDIportal [36], which provide comprehensive resources for observable RNA editing events in the human transcriptome. Over the last decade, many studies have systematically compared genome-wide editing profiles between cancer and normal samples in different cancer types by using RNA-Seq data from a large number of samples [37,38,39,40]. Their results further indicate that A-to-I editing plays a role in the formation, progression and molecular identification of cancer, and may provide new insight into the development of novel diagnostic and prognostic markers and new therapy methods for cancer patients.

Here, we provide a genome-wide investigation of A-to-I RNA editing in Non-Hodgkin lymphomas (NHLs, cancers of B, T or natural killer lymphocytes) using a large number of NHL and normal samples. While a recent study on RNA editing in NHL [41] has focused on finding specific mechanistically relevant editing events, our emphasis here is more on the question of RNA editing levels as a biomarker in NHL. We systematically investigate A-to-I RNA editing in NHL by analyzing editing profiles containing only previously annotated A-to-I editing events, using high-throughput RNA sequencing data from Morin et al. [42]. RNA sequencing data of normal B-cell samples from separate studies [29,43] are used as normal controls. We determine known A-to-I editing events from a public database that show significant differences between NHL and normal samples (or between samples from different NHL subtypes). Our results show that a number of known editing sites are differentially edited, most of which are located in the non-coding regions with no preference for the direction of editing changes, and that many sites located in the UTRs show a strong correlation between gene expression level and editing efficiency, suggesting a functional relationship. Additionally, using a leave-one-out cross-validation method and unsupervised clustering of the samples, we establish that genome-wide RNA editing profiles alone contain sufficient information to differentiate NHL from normal samples and between different NHL subtypes. These results confirm that NHL and RNA editing are intimately linked.

## 2. Results

### 2.1. Workflow

Figure S1 shows the workflow which we follow in our analysis (for details see Section 4). In short, we first align RNA-Seq reads from NHL and normal samples against the human genome. Once suitable RNA-Seq alignments are generated, we count read coverage similarly to the approach of REDItoolKnown.py [44] for each known editing site included in the RADAR database. Then, we filter sites based on multiple filters with stringent thresholds and calculate the editing efficiency for the sites, which pass all filters. These steps create an editing profile for each sample. Then, we apply statistical approaches to make comparisons for each editing site and identify all known editing sites showing significant differences between groups of samples. We want to emphasize that while there are many tools such as REDItools [44], GIREMI [45], JACUSA2 [46], RNAEditor [47] and FLARE [48] that focus on the de novo discovery of novel editing sites, we here take the more conservative approach to only quantify the editing level at *known* editing sites and do not attempt to discover entirely novel editing sites.

### 2.2. A Number of Sites Display Differential Editing among Different Subtypes in NHL

**Figure 1:**
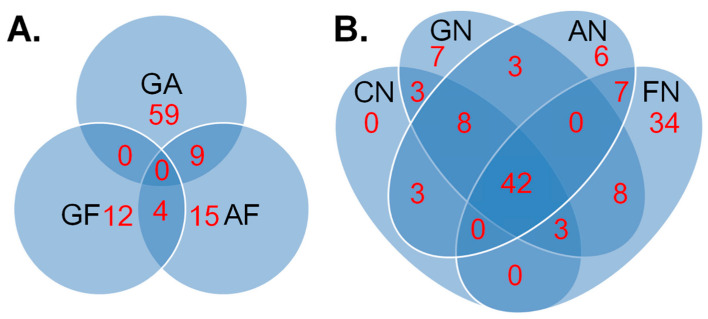
Differentially edited site counts in comparisons between (**A**) NHL subtypes and (**B**) NHL and Normal. “GA” represents the comparison between GCB and ABC, “GF” represents the comparison between GCB and FL, “AF” represents the comparison between ABC and FL, “CN” represents the comparison between all NHL samples and Normal, “GN” represents the comparison between GCB and Normal, “AN” represents the comparison between ABC and Normal, and “FN” represents the comparison between FL and Normal. The numbers show how many differentially edited sites are located in each category.

We first compare editing profiles between NHL subtypes to identify any known editing events that are differentially edited among different NHL subtypes. The 99 Non-Hodgkin lymphoma (NHL) samples can be classified into three subtypes: 54 samples are germinal center B-cell (GCB), 32 samples are activated B-cell (ABC) and 13 samples are follicular lymphoma (FL) [42]. We follow the workflow (Figure S1) and filter sites based on read coverage, editing efficiency, SNPs and other criteria (details see Section 4). We perform three comparisons between these NHL samples: ABC vs. FL, GCB vs. FL and GCB vs. ABC. We apply statistical approaches which take into account the read coverages from different samples (the statistical uncertainty of editing efficiency is different in samples with different read coverages, details see Section 4) to compare editing levels at sites that passed the filters and identify all editing sites showing significant differences in editing level at a cutoff of a false discovery rate [49] of 0.05 in a comparison. As shown in Table 1, Figure 1A and Table S1, among hundreds of sites that pass the filters (Table S2), only a few editing sites are differentially edited in different NHL subtypes. Most of these differentially edited sites are located in the 3′UTRs, while a few sites are located in the introns, intergenic regions and 5′UTRs (Table 1). For each comparison, some of the differentially edited sites display hypo-editing, while others display hyper-editing (Table 1, column 3), indicating that the differences cannot be simply explained by global differences in ADAR activity. Moreover, we note that several differentially edited sites are located in genes that are known to be cancer-related (Table 1), such as cathepsin S (CTSS), cathepsin B (CTSB) and protein kinase C substrate 80K-H (PRKCSH), NOP14 nucleolar protein (NOP14), serine/threonine kinase 4 (STK4), von Hippel–Lindau tumor suppressor, E3 ubiquitin protein ligase (VHL), and SAM domain and HD domain 1 (SAMHD1). CTSS mutations (but not editing) have even explicitly been shown to be tumor promoting in follicular lymphoma [50].

### 2.3. More Sites Are Differentially Edited between NHL and Normal

Next, we compare editing profiles between NHL and normal samples. We apply the workflow to RNA-Seq data from 41 normal B-cell [29,43] samples and filter editing sites based on the same criteria as for the NHL subtypes. Then, we perform four comparisons between NHL and normal samples: NHL (all 99 samples) vs. Normal, GCB vs. Normal, ABC vs. Normal and FL vs. Normal. Similar to the results in the NHL subtype comparisons, hundreds of sites pass the filters, but more sites than for the NHL subtype comparison with much higher statistically significant differences are differentially edited (Table 2, Figure 1B and Table S1). This indicates that editing profiles between NHL subtypes are more similar than editing profiles between NHL and normal samples. Similar to the comparison between NHL subtypes, we find that most of the differentially edited sites are located in UTRs, introns, and intergenic regions (Table 2). However, we also find two editing sites in coding regions, both of which are non-synonymous recoding events (Table 2). Specifically, one recoding site located in AZIN1 has previously shown to be differentially edited between hepatocellular carcinoma [18], non-small-cell lung cancers [19], esophageal squamous cell carcinoma [20], and colorectal cancer [21,22,23], and their respective normal tissues. More interestingly, a Glu-to-Gly recoding event in the PRKCSH gene has to our knowledge not been reported before in the context of cancer even though the gene itself is known as a potential cancer biomarker [51]. Again, the direction of the changes in editing efficiency is not consistent with global changes in ADAR activity. We again find several sites located in genes that are known to be cancer-related, such as cathepsin S (CTSS), protein kinase C substrate 80K-H (PRKCSH), serine/threonine kinase 4 (STK4), antizyme inhibitor 1 (AZIN1), NOP14 nucleolar protein (NOP14), von Hippel–Lindau tumor suppressor, E3 ubiquitin protein ligase (VHL) and tumor protein p53 (TP53).

### 2.4. Gene Expression Is Highly Correlated with Editing Efficiency of Differentially Edited Sites in UTRs

**Figure 2:**
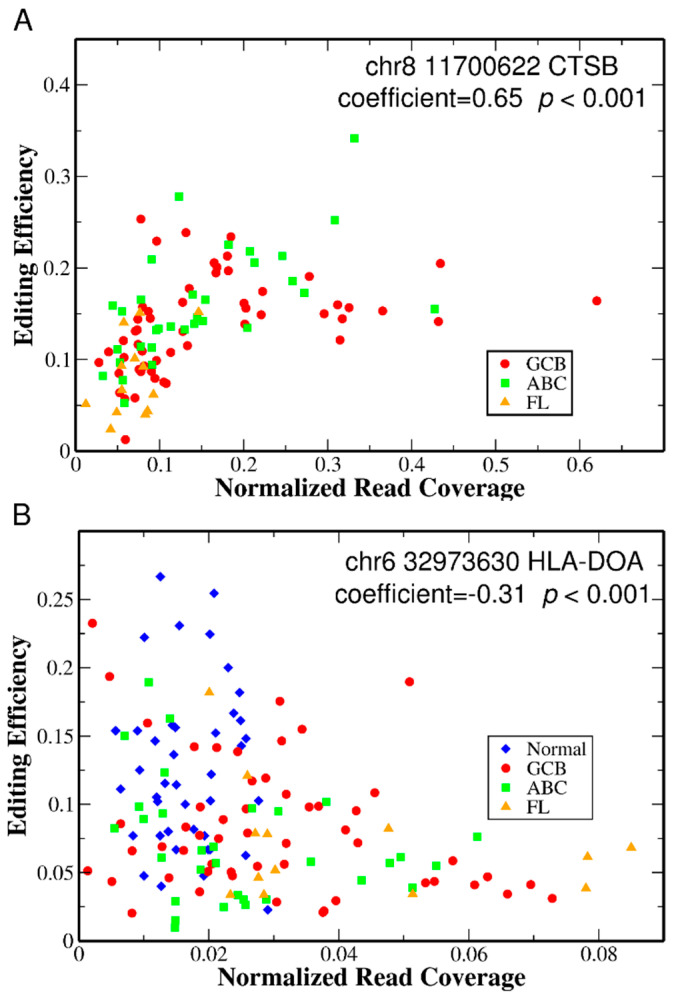
Correlation plots of editing efficiency and gene expression for two differentially edited sites with examples of (**A**) positive and (**B**) negative correlations. The different symbols represent the different sample groups. (**A**). Editing efficiency and normalized read coverage of the entire CTSB gene for one differentially edited site (chromosome 8 at position 11700622) from the ABC vs. FL comparison group among all 99 NHL samples (GCB + ABC + FL). The read coverage for this specific site is too low to pass the minimum coverage filter, as mentioned in Section 4, in all 41 normal samples. Thus, no data for normal samples are shown. The Spearman’s rank correlation coefficient is 0.65 using the cor.test function in R, with an FDR-adjusted *p*-value < 0.001. (**B**). Editing efficiency and normalized read coverage of the entire HLA-DOA gene for one differentially edited site (chromosome 6 at position 32973630) from all 140 samples. The Spearman’s rank correlation coefficient is −0.31 using the cor.test function in R, with an FDR-adjusted *p*-value < 0.001.

A-to-I editing in non-coding regions has the potential to affect mRNA stability, splicing and nuclear retention [3,4]. We thus ask if each of the differentially edited sites in UTRs have a potential role in the aberrant gene regulation characteristic of a cancer cell by correlating gene expression levels with editing efficiencies. The rationale is that for each of the differentially edited sites in UTRs, if they play a role in nuclear retention or affect mRNA stability, the gene expression level should be correlated with their editing efficiency. For example, for editing in CTSS, which we found above to be differentially edited, a mechanism for expression level control by an editing site has been documented [52]. At each given editing site, we examine the correlation between editing efficiency and corresponding gene expression measured by the normalized read coverage of the entire gene for each patient using Spearman’s rank correlation coefficients, and determine statistical significance (details see Section 4.6). For all the differentially edited sites in UTRs from all the seven comparisons above, we find that 39 out of 88 tested sites show a significant correlation (FDR multiple testing correction with adjusted *p*-value cutoff of 0.05) between editing efficiency and gene expression level (Table 3). Most of these sites show a positive correlation (32/39), while very few sites show a negative correlation (7/39). Typical correlation plots of editing efficiency and gene expression from two different genes are shown in Figure 2.

### 2.5. The Clinical Status of Samples Is Predicted with High Accuracy Based on RNA Editing Profiles Alone

To further test the link between A-to-I editing and NHL, we ask the question of whether we can make predictions for samples with unknown clinical status just based on genome-wide RNA editing profiles. To address this question, we use leave-one-out cross-validation (details see Section 4.7). Specifically, for each comparison, we use one sample as the validation data (assuming the clinical type is “unknown”) and the remaining observations as the training data (the type is “known”: NHL or Normal or GCB or ABC or FL). Then, we perform the same comparison analysis for the training data as described in the last two sections, which results in a number of sites showing differential editing. The comparison information (average editing efficiency at each site, for details see Section 4) from the training data is then used to determine the status of the validation data. This step in the comparison is then repeated for all the samples to determine the type of every sample based on the comparison information from the other samples. The predicted “type” of each sample is then compared to the real “type” of the sample. Table 4 shows the validation results from all seven groups we compare. For each comparison, we correctly predict the status for most of the samples (Fisher’s exact test shows that all the results have highly significant *p*-values), indicating that the A-to-I editing profile alone is able to discriminate NHL and normal samples as well as NHL subtypes. As shown in Table 4, both prediction accuracy and *p*-value are more significant when comparing NHL samples with normal samples than for comparisons of NHL subtypes, suggesting that editing profiles among NHL subtypes are more similar than editing profiles between NHL and normal samples.

### 2.6. Unsupervised Clustering Can Differentiate NHL and Normal Samples but Not NHL Subtypes

**Figure 3:**
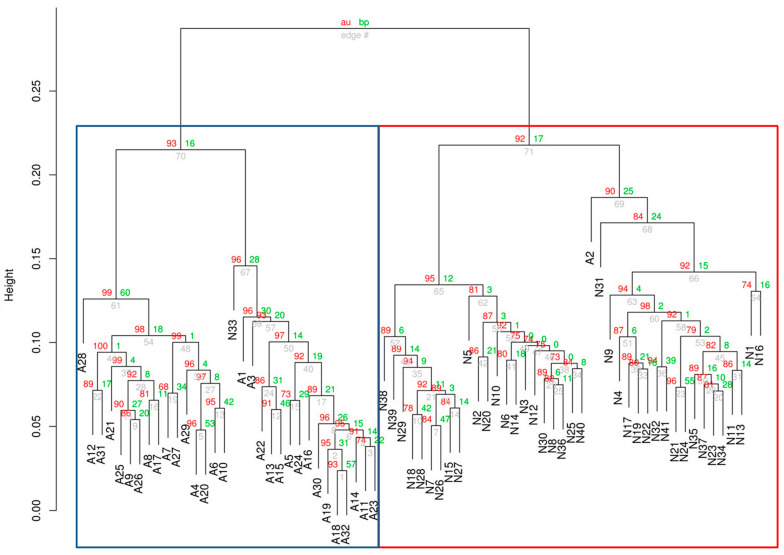
Hierarchical clustering dendrogram of A-to-I RNA editing among ABC and normal samples. Clustering was performed with the R package *pvclust*. Values at branches represent multiscale bootstrap-calculated approximately unbiased (AU) *p*-values and bootstrap *p*-values. Cluster labels indicating group membership (the real status of the samples: Ai, i = 1, 2, …, 32; Nj, j = 1, 2, …, 41) are shown below the branches. Membership in the two largest clusters is tracked in the left and right box, which match the NHL (ABC, Ai) and Normal (Nj) groups well. The Spearman’s rank correlation coefficient is −0.31 using the cor.test function in R, with an FDR-adjusted *p*-value < 0.001.

**Figure 4:**
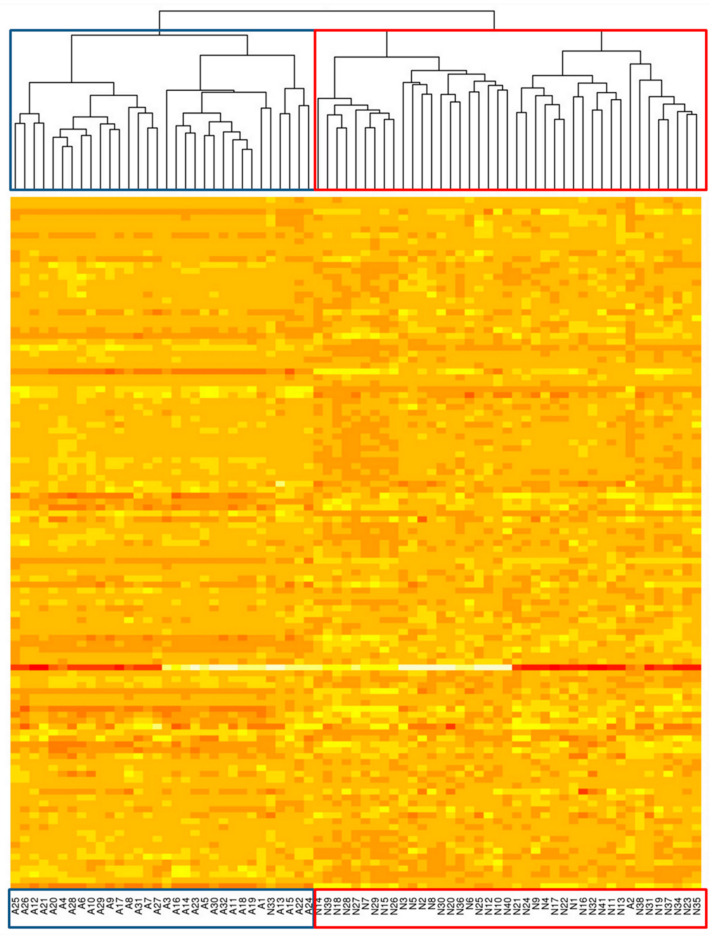
Full hierarchical clustering dendrogram and heatmap of A-to-I RNA editing among ABC and normal samples generated in R with the *pvclust* package.

To further investigate the ability of RNA editing profiles to distinguish sample types, we apply unsupervised analysis to cluster all samples into subgroups based on their genome-wide editing profiles. We test this idea on four groups of samples (All NHLs + Normal; GCB + Normal; ABC + Normal; and FL + Normal). In each group, we follow the workflow and filter editing sites based on read coverage, editing efficiency, SNPs and other criteria (for details see Section 4). All the sites passing the filter are used in clustering. The set of selected editing sites and thus the clustering results differ from comparison to comparison, but we emphasize that none of the filters uses the “labelling” of the samples. Samples are ordered according to the hierarchical clustering results of their genome-wide A-to-I editing profiles (for details see Section 4.8); thus, highly correlated patients who share similar editing profiles are located next to each other. As shown in Figure 3, Figure 4 and Figures S2–S4, the four groups can be successfully separated into two clusters each and each cluster matches well with the Normal and the NHL group. These results further support that A-to-I editing is closely linked to NHL. We do not observe clear clusters for unsupervised clustering of only NHL samples (for different subtypes); this is probably due to the fact that the genome-wide editing profiles for NHL subtypes are much more similar to each other compared to the differences between NHL and normal samples.

## 3. Discussion and Conclusions

While several studies show that A-to-I editing in several individual genes is differentially regulated in cancer and normal samples and analyze transcriptome-wide A-to-I editing profiles in a limited number of samples, several recent works provide transcriptome-wide characterization of A-to-I editing in different cancer types at a large scale [37,38,39,40]. In this work, we provide a genome-wide investigation of A-to-I RNA editing in NHL using a large number of samples that focuses on the ability to distinguish cancer subtypes from their global editing profiles. We systematically investigate A-to-I RNA editing in NHL by analyzing editing profiles containing known A-to-I editing events using cancer RNA-Seq data from Morin et al. [42] paired with RNA-Seq data of normal B-cell samples from Li et al. [29] and Toung et al. [43] as normal controls.

We note that several genes in our list of differentially edited sites between NHL and Normal, including AZIN1 (antizyme inhibitor 1), MAGT1 (magnesium transporter 1) and PAICS (phosphoribosylaminoimidazole carboxylase, phosphoribosylaminoimidazole succinocarboxamide synthetase), have been shown to be differentially edited between HCC and normal tissue in previous studies as well [18,53]. Also in agreement with one of these previous HCC studies [53], we find that most of the differentially edited sites are located in non-coding regions and that there is no general trend for these sites, i.e., some of the sites show hypo-editing in NHL, while the others show hyper-editing in NHL (see Table 1 and Table 2). The absence of a preference for hyper-editing in NHL samples indicates that these abnormal A-to-I editing events are not simply a stress response. In addition, we notice that many of these genes contain multiple significantly differentially edited sites pointing toward processivity of the mechanism responsible for the differential editing. Interestingly, the genes found by us are different from the genes found to be prominently edited in a previous NHL RNA editing study [41], namely ATM (ataxia telangiectasia mutated), MDM4 (homolog of mouse double minute 4), and MAVS (mitochondrial antiviral signaling protein). We find that a number of the differentially edited sites in UTRs show strong correlations between editing efficiency and gene expression level, indicating that they may affect mRNA stability or nuclear retention and thus directly contribute to aberrant gene regulation. Other sites tested, which do not exhibit strong correlation, may still have biological outcomes since multiple edited sites in one gene may contribute to the gene expression level cooperatively and their functions may not be independent, thus masking the biological effect when looking at one site at a time. These sites might also affect translation efficiency which we are not able to probe.

In our analysis, we removed duplicate reads and only kept the read with the highest base quality (see Section 4), since the former may result from amplification artifacts in the PCR process during library construction. However, duplicate reads may also result from high expression levels of individual genes and it is difficult to determine whether a duplicated read is an artefact or not. We thus performed our analysis again with all the same criteria (as shown in Section 4), except that we did not remove duplicated reads. The comparison results of genome-wide editing profiles (between NHL subtypes as well as between NHL and Normal) are qualitatively similar to the case where we remove the duplicate reads (Table S3) (they have a similar number of differentially edited sites, more than half of the sites are identical and the sites that do not agree all stem from cases were one of the criteria, such as *p*-value, coverage, or average editing efficiency, is close to our cutoffs). This indicates that our analysis is robust with respect to duplicate read removal.

While it is tempting to suspect that differences in editing activity between groups might be driven by systematic differences in expression levels of ADAR1 and/or ADAR2 between groups, this is inconsistent with the fact that we see significant changes in editing levels in both directions in the same comparisons. We also explicitly calculated Spearman’s rank correlations of the editing levels with ADAR1 and ADAR2 expression for the editing sites in Table S3 using the same approach as for the correlations between editing efficiency and expression of nearby genes. We found that 148 of 696 calculated correlations with either ADAR1 or ADAR2 expression level are statistically significant after Benjamini Hochberg correction (corresponding to an uncorrected *p*-value of less than 0.0105). This indicates that while the majority of the significant differences we observe in editing are not driven by ADAR levels, about a quarter might be.

Our workflow provides an extensive analysis of A-to-I editing in NHL and reveals a sizeable number of known editing sites that are differentially edited. However, due to our rather conservative approach, there is the potential that some differentially edited sites have been missed by our analysis. In the past, a lot of controversies have been raised about the discovery of new editing sites [54,55,56,57]; thus, to be conservative, we are not looking for new editing sites but rather limit ourselves to those deposited into the RADAR database. As a result, some potential novel differentially edited sites may be missing from our list. Moreover, in order to reduce statistical error when quantifying editing efficiency, we require that an editing site is covered by at least 10 reads in every sample (see Section 4.2); this may also result in missed editing sites that are expressed at low levels. Furthermore, we do not consider the effect of copy number variations (CNVs) in NHL, a form of structural variation which is common in cancer genomes, which, in combination with SNPs, may affect our editing efficiency calculations and editing site calling. In addition, we notice that our RNA-Seq data for Non-Hodgkin lymphomas (NHLs) [42] and normal B-cell samples [29,43] are from two different studies, which were created using different library preparation protocols; thus, batch effects between libraries might influence our comparison results between NHL and normal samples, but do not affect comparison results between NHL subtypes.

The removal of SNPs is an essential component of our workflow. We filtered SNPs by using relatively stringent criteria directly applied to our data (described in Section 4.4) instead of using the NCBI dbSNP database [58] due to the fact that some SNPs are missing from the dbSNP database and more importantly a number of sites that are included in the dbSNP database are not real SNPs but A-to-I editing sites added to the dbSNP database based on cDNA evidence [59,60]. While we cannot rule out the possibility that some rare SNPs are still included in our list, fewer than 10 SNPs are removed by our SNP filter in all comparisons, and if we very conservatively assume the rate of real SNPs that are not removed by our SNP filter to be 10%, at most 1 SNP will pass the filter and be in our final list; thus, given the total numbers of identified differentially edited sites (Table 1 and Table 2, at least 13 for all comparison groups), we believe that undetected SNPs are not a major issue affecting our results.

In this study, we systematically investigated A-to-I RNA editing in NHL by analyzing editing profiles containing known A-to-I editing events using publicly available RNA sequencing data. We identified a number of known editing sites from a public database showing significant differences between NHL and normal samples (and between samples from different NHL subtypes). Most of these sites are located in non-coding regions with no preference for hyper-editing in NHL, and several genes show a strong correlation between gene expression level and editing efficiency, may suggest potential biological functions. Furthermore, we showed that editing profiles alone contain sufficient information to distinguish NHL from normal samples and even different NHL subtypes, thus confirming a strong connection between RNA editing and NHL. The fact that RNA editing alone can be used to differentiate between NHL subtypes indicates that RNA editing efficiency should be incorporated into molecular cancer biomarkers. Future studies could investigate the correlation between RNA editing and NHL stages to probe the possibility of its use in staging. Moreover, the genes, which we have identified as being regulated by significantly differentially editing sites, and especially the novel Glu-to-Gly alteration in the PRKCSH gene caused by an editing site that is significantly differentially edited between normal samples and every cancer group we looked at in this study, warrant further study to determine their potential as therapeutic targets for NHL.

## 4. Materials and Methods

### 4.1. Mapping RNA-Seq Reads to the Reference

We obtained RNA-Seq data for 99 Non-Hodgkin lymphomas (NHLs) from Morin et al. [42] and for normal B-cells from Li et al. [29] and Toung et al. [43] (for accession numbers and subtypes see Table S4). For each sample, we mapped the RNA-Seq reads against the hg19 reference genome using STAR (Spliced Transcripts Alignment to Reference) [61]. Most parameters in the alignment process were set to their default values. Since most of the RNA-Seq data are paired-end, we chose paired-end options in STAR. The mapping output was set to SAM format [62] resulting in the mapping command “STAR −−genomeDir −−readFilesIn fastq1 fastq2 −−runThreadN 4 −−outFileNamePrefix −−genomeLoad LoadAndKeep −−outSAMunmapped Within −−outSAMstrandField intronMotif”. The output SAM files were first converted to their binary versions (BAM files) and then these BAM files were sorted and indexed for rapid lookup using SAMtools [62].

### 4.2. Filtering, Editing Site Selection, and Editing Efficiency

We downloaded the list of all A-to-I editing sites in the RADAR RNA editing database (http://RNAedit.com, accessed on 17 January 2016) [35]. As the RADAR RNA editing database has become defunct since we embarked on this analysis, we provide the full list of editing sites used in Table S5 and also include links to the more up-to-date REDIportal database [36] for the editing sites in our results tables.

For each site in the list, we counted the number of reads that could be mapped to this site for every sample. To accurately count the read coverage at each site and eliminate false positives, several filters were applied following the order described below:

1. We removed duplicate reads (defined as reads having the exact same sequence with their mate and mapping to the same position in the reference), and kept the read with the highest base quality;
2. To ensure the mapping uniqueness of a read, we only counted reads with a mapping quality score of at least 10;
3. We discarded a read if the editing position was within 2 bp of the 5′ or 3′ end;
4. We only counted a read if the editing site of the read had a base quality score of at least 20.

After this read counting step, a list of read coverages at each editing site in the database for each sample was created. To ensure sufficient statistical power for each comparison, we only selected editing sites in which every sample had coverage of at least 10 reads. For the selected editing sites, using the same filters as described above, we further counted the number of reads that showed base “G” (*n_I_*, edited) and the number of reads that showed base “A” (*n_A_*, unedited) at each editing site in the forward strand and counted the number of reads that showed base “C” (*n_I_*, edited) and the number of reads that showed base “T” (*n_A_*, unedited) at each editing site in the reverse strand. We further denoted the total number of “A” and “G”, or “T” and “C” as *n*. Finally, for each selected site, the editing efficiency was determined as *n_I_/(n_I_ + n_A_) = n_I_/n*.

### 4.3. Grouping Samples

Based on the clinical data, all the samples were assigned to 5 groups (see Table 5). Among these five groups, we performed seven comparisons, namely, GCB vs. FL, GCB vs. ABC, ABC vs. FL, GCB vs. Normal, ABC vs. Normal, FL vs. Normal and NHL vs. Normal.

### 4.4. Further Filtering of Editing Sites for Statistical Comparison

For each site, if the *average* editing efficiency in both groups of a comparison pair was very low (below 0.05) or very high (above 0.95), the site was removed. Furthermore, we denoted an editing site as a potential SNP and also removed it from further analysis based on the following criteria (determined from the editing efficiency data for all the samples in a comparison): for each editing site *within one sample*, we obtained an editing efficiency. We denoted the number of samples showing an editing efficiency at a given site between 0 and 0.1 as *m_AA_*, between 0.4 and 0.6 as *m_AG_*, and between 0.9 and 1 as *m_GG_*, and we called the total number of samples M. Then, a putative SNP was an editing site which fulfilled the following two conditions, and was removed from the list:

1. *(m_AA_ + m_AG_ + m_GG_)/M* > 40% (a significant number of samples showed editing efficiencies between 0 and 0.1, 0.4 and 0.6, or 0.9 and 1, consistent with homozygotic or heterozygotic SNPs);
2. At least two of the three conditions *m_AA_/M* > 5%, *m_AG_/M* > 5%, and *m_GG_/M* > 5% were satisfied (to ensure that there was variation between the configurations of an SNP in the sample population).

### 4.5. Statistical Comparison of Groups

For each editing site, due to the variation in read coverage for each sample, the statistical uncertainty of editing efficiency for each sample was different. Samples with higher depth should be less uncertain than samples with lower depth in editing efficiency. Thus, samples with higher depth should contribute more to statistical analysis than samples with lower depth in one group. To address this problem, we introduced a weight vector for each site in an unpaired two-tailed Student’s *t*-test. Since the *t*-test is a special case of simple linear regression (when the independent variable is dichotomous with two points 0 and 1), we thus performed a weighted linear regression, with weights *w_i_ =* 1/*σ_i_^2^*, where *σ_i_* is the standard error of the editing efficiency of sample *i* calculated as follows:

For each editing efficiency derived from *n_I_/n*, we assumed that *n_I_* follows the binomial distribution with parameters *n* and *p*, that is, *n_I_ ~ B(n*, *p)*, where *p = n_I_/n* (the estimated editing efficiency value). Thus, we had (note: <*x*> represents the average of *x*)

(1) $$<\left(\frac{n_{I}}{n}\right)>=p$$

(2) $$<\left(\frac{n_{I}(n_{I}-1)}{n}\right)>=\left(n-1\right)p^{2}.$$

Based on the two equations above, we obtained

(3) $$<\left(\frac{n_{I}^{2}}{n}\right)>=\left(n-1\right)p^{2}+p.$$

Therefore,

(4) $$\sigma^{2}\left(\frac{n_{I}}{n}\right)=<{\left(\frac{n_{I}}{n}\right)}^{2}>-{<\left(\frac{n_{I}}{n}\right)>}^{2}=\frac{1}{n}p\left(1-p\right)=\frac{n_{I}n_{A}}{n^{3}}$$

and we obtained the weighted score for a sample as

(5) w=1σ2=n3nInA.

We note that our approach addresses the variability in sequence coverage from sample to sample by assuming a binomial distribution similar to the REDIT tool [63], but differs from REDIT in that the test for differential editing is a weighted *t*-test rather than a maximum likelihood estimation using β distributions.

### 4.6. Correlating Editing Efficiency with Gene Expression

We selected all the differentially edited sites located in UTRs from all the 7 comparisons (88 sites), identified the 27 genes they are located in and calculated the normalized read coverage of these genes for each patient. The normalized read coverage of all the genes was calculated based on the number of reads mapped to the exonic region of each gene divided by the number of all mapped reads for that sample. For all 88 sites, we then plotted their editing efficiencies against the normalized read coverages of their containing gene for each patient (but excluded the samples for which the read coverage at the differentially edited sites was below 10). The correlation was quantified by Spearman’s rank correlation coefficient and statistical significance was determined using the *cor.test* function in R. The alternative hypothesis was set to “greater” if the Spearman’s rank correlation coefficient was greater than zero and to “less” if the Spearman’s rank correlation coefficient was less than zero.

### 4.7. Leave-One-Out cross Validation

For each comparison (N samples, two groups—Group I and II), one sample was selected as the validation sample (with the type assumed “unknown”) and the remaining (N − 1) samples were used as the training data. We used the same statistical comparison method as described above for the (N − 1) samples. The sites showing significantly differential editing (FDR multiple testing correction with adjusted *p*-value of 0.05) were selected, and the average editing efficiencies at each site for the two comparison groups ((N − 1) samples) were then used to determine the status of the validation data. For these selected sites, we counted read coverage and calculated editing efficiency for the validation sample using the method described above. Then, we scored the validation sample based on read coverage and editing efficiency for each of the selected sites as follows:

- If read coverage was less than 10, we scored the site as “0”;
- If read coverage was at least 10 and editing efficiency was closer to the mean of Group I than to the mean of Group II, we scored the site as “1”;
- If read coverage was at least 10 and editing efficiency was closer to the mean of Group II than to the mean of Group I, we scored the site as “−1”;

We added the scores of all selected sites and obtained a final score for the validation sample. If the final score was greater than zero, we assigned the sample to Group I. If the final score was less than zero, we assigned the sample to Group II. If the final score was equal to zero, we declared the sample as “Not Determined”. We then repeated these steps for all the samples in a comparison and evaluated the prediction accuracy for each comparison.

The statistical significances of the results of the leave-one-out cross-validation were evaluated by Fisher’s exact test. For each comparison group, we created a 2 × 2 contingency table which represents the number of samples located in each category, where the categories are split by prediction of being in Group I or II and actually being in Group I or II. *p*-values were then computed by Fisher’s exact test.

### 4.8. Clustering

To further investigate if A-to-I editing can be used as a classifier in NHL, unsupervised analysis was applied to cluster all samples into several subgroups based on the editing levels of sites. All the samples were assumed to be of unknown type and clustering of the data was a workflow that took a data matrix of the samples and the editing efficiency value of each selected site for a given set of samples. Editing sites were selected based on the following criteria: we selected editing sites in which every sample had coverage of at least 10 reads, removed SNPs based on the criteria described above and calculated the average editing efficiency (weighted) for all the samples and discarded the sites with average editing efficiency below 0.02 or above 0.98. We used R for matrix manipulation and *pvclust* implemented in R for unsupervised clustering based on the editing efficiencies of the remaining sites. Adjusted *p*-values were obtained via multiscale bootstrap resampling of the data. Our clustering workflow used the Pearson correlation distance measure and the “Average” clustering method. Both dendrograms and heatmaps were produced to visualize the relationship between the clustering sample members.

For better visualization of the heatmap, we rescaled the editing efficiency for each selected editing site, using the average editing efficiency calculated from all the samples; for each site, we first calculated the average editing efficiency across all the samples and then subtracted the average editing efficiency from the original editing efficiency for each sample, resulting in a normalized editing efficiency value and creating a normalized data matrix, which consisted of both negative and positive values. The rescaled values replaced the raw editing efficiency data and were used to create heatmaps.

## Figures and Tables

**Table 1:** Comparisons between NHL subtypes ^1^.

Comparison of Groups	Number of Sites Tested	Number of Sites with Significant Differences (FDR *p*-Value < 0.05)	Known Cancer-Related Genes	Number of Sites in Each Category			
				UTRs	Introns	Intergenic Regions	Repetitive Elements
ABC vs. FL	543	28 (16/12) ^2^	CTSS, CTSB, STK4, SAMHD1	21	4	3	25
GCB vs. FL	546	16 (14/2) ^2^	CTSS, CTSB, PRKCSH	14	2	0	15
GCB vs. ABC	502	68 (62/6) ^2^	NOP14, SAMHD1, VHL	30	21	15	67

^1^ See Table S1 for a detailed list of all editing sites, genes and *p*-values. ^2^ (x/y): x represents the number of the differentially edited sites displaying a higher average editing efficiency in the first group than in the second group (number of sites that are hypo-editing in the second group); y represents the number of the differentially edited sites displaying a higher average editing efficiency in the second group than in the first group (number of sites that are hyper-editing in the second group).

**Table 2:** Comparisons between NHL and Normal ^1^.

Comparison of Groups	Number of Sites Tested	Number of Sites with Significant Differences (FDR *p*-Value < 0.05)	Known Cancer Related Genes	Number of Sites in Each Category				
				Coding Regions	UTRs	Introns	Intergenic Regions	Repetitive Elements
NHL vs. Normal	398	59 (18/41) ^2^	STK4, AZIN1, CTSS, NOP14, PRKCSH	2	22	28	7	56
GCB vs. Normal	464	74 (19/55) ^2^	STK4, AZIN1, CTSS, NOP14, PRKCSH, VHL, TP53	2	27	35	12	71
ABC vs. Normal	484	69 (20/49) ^2^	STK4, AZIN1, CTSS, PRKCSH, VHL	2	25	33	9	66
FL vs. Normal	496	84 (32/52) ^2^	AZIN1, CTSS, PRKCSH, VHL, TP53	2	35	35	12	79

^1^ See Table S1 for a detailed list of all editing sites, genes, and *p*-values. ^2^ (x/y): x represents the number of the differentially edited sites displaying a higher average editing efficiency in the first group than in the second group (number of sites that are hypo-editing in the second group); y represents the number of the differentially edited sites displaying a higher average editing efficiency in the second group than in the first group (number of sites that are hyper-editing in the second group).

**Table 3:** Correlation between editing efficiency and gene expression for differentially edited sites in UTRs.

Number of Sites Tested	Significant Correlation (FDR *p*-Value < 0.05)	Positive Correlation	Negative Correlation
88	39	32	7

**Table 4:** Performance of leave-one-out cross-validation.

Groups	Total Number of Samples	Number of Samples Correctly Predicted	*p*-Value (Fisher’s Exact Test)
ABC vs. FL	45	38 (84%)	2.50 × 10^−5^
GCB vs. FL	67	57 (85%)	2.41 × 10^−6^
GCB vs. ABC	86	65 (76%)	4.54 × 10^−5^
NHL vs. Normal	140	130 (93%)	4.35 × 10^−25^
GCB vs. Normal	95	86 (91%)	1.56 × 10^−17^
ABC vs. Normal	73	70 (96%)	5.26 × 10^−17^
FL vs. Normal	54	53 (98%)	1.26 × 10^−11^

**Table 5:** Grouping of samples by clinical status.

Clinical Status	Number of Samples
GCB	54
ABC	32
FL	13
Normal	41
NHL (GCB + ABC + FL)	99

## Data Availability

Restrictions apply to the availability of these data. The results published here are in whole or part based upon data generated by the Cancer Genome Characterization Initiative (phs000235), Non-Hodgkin Lymphoma project, developed by the NCI. The data used for this analysis are available at http://www.ncbi.nlm.nih.gov/projects/gap/cgi-bin/study.cgi?study_id=phs000235.v6.p1 (accessed on 30 January 2013). Information about CGCI projects can be found at https://ocg.cancer.gov/programs/cgci (accessed on 30 January 2013).

## Supplementary Materials

The following supporting information can be downloaded at: https://www.mdpi.com/article/10.3390/genes15070864/s1, Table S1: Data for individual editing sites for each comparison. Figures S1–S4, Tables S2 and S4: Collection of supplementary figures and tables. Table S3: Data for individual editing sites for each comparison when analyzing without removal of duplicate reads. Table S5: List of all editing sites used in the analyses.
