# Supplementary material for: A-to-I Editing Is Subtype-Specific in Non-Hodgkin Lymphomas
Source: Genes (Basel). 2024 Jul 1;15(7):864. doi: 10.3390/genes15070864 (PMC11276283; doi:10.3390/genes15070864)
Supplement: Supplementary file 1 [file genes-15-00864-s001.zip › A-to-I editing Supplementary Material.pdf]

Supplementary Figures

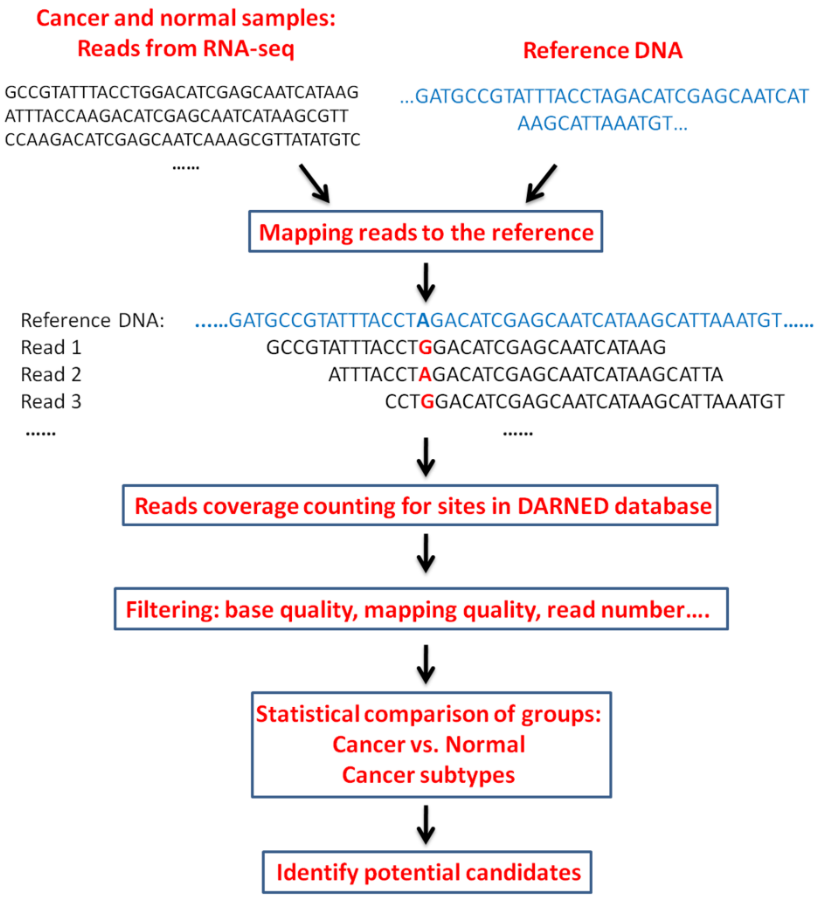

Figure S1. General workflow to determine A-to-I editing events that show significant differences between sample groups.

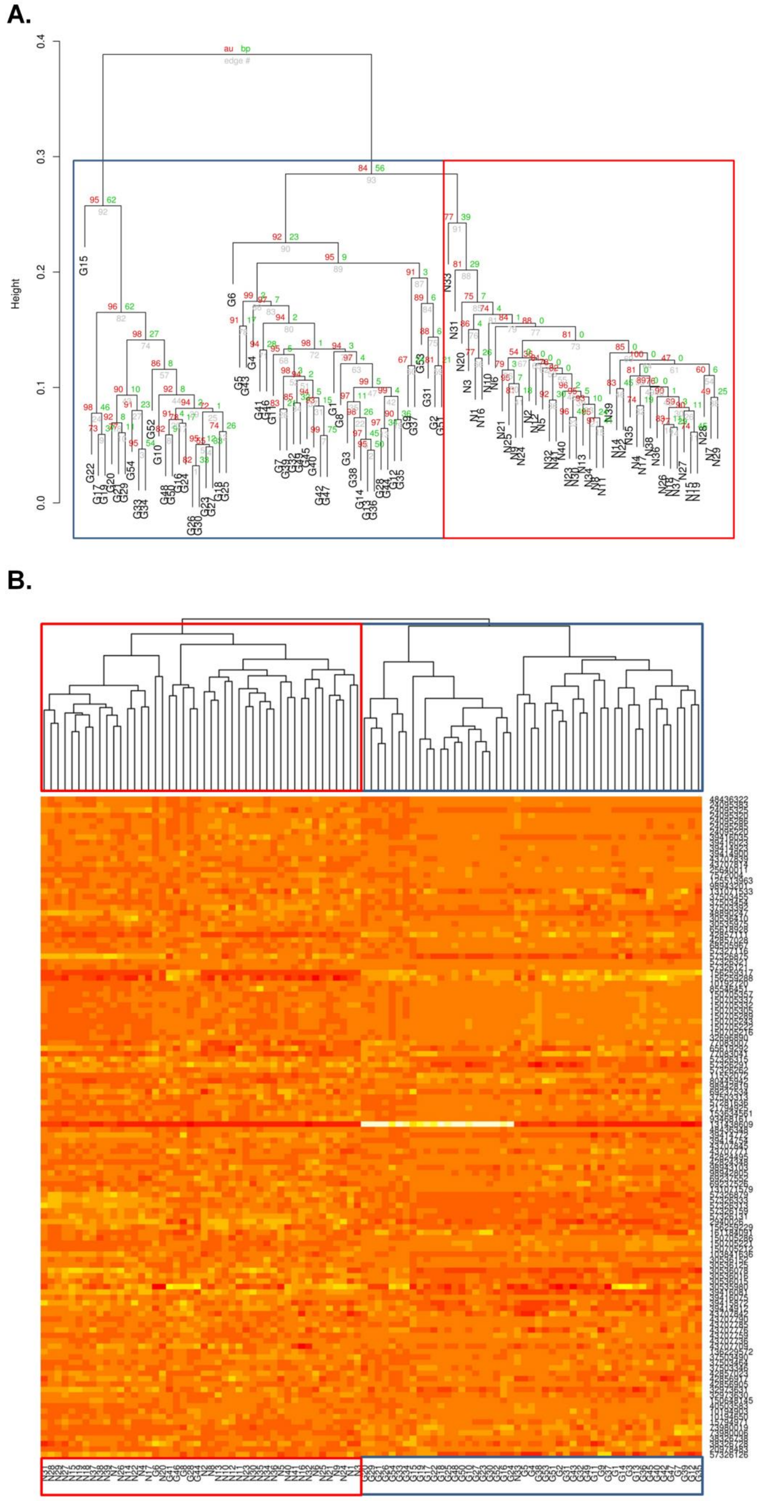

Figure S2. Hierarchical clustering (A) dendrogram and (B) heatmap of A-to-I RNA editing among GCB and Normal samples. Clustering was performed with the R package *pvclust*. Values at branches represent multiscale bootstrapping calculated approximately unbiased (AU) *p*-values (red) and bootstrap *p*-values (green). Cluster labels indicating group membership (the real status of the samples: Gi, i=1,2,...54; Nj, j=1,2,...41) are shown below the branches. Membership in the two largest clusters is tracked in the blue (left cluster) and red (right cluster) box, which match the NHL (GCB, Ai) and normal (Nj) groups well.

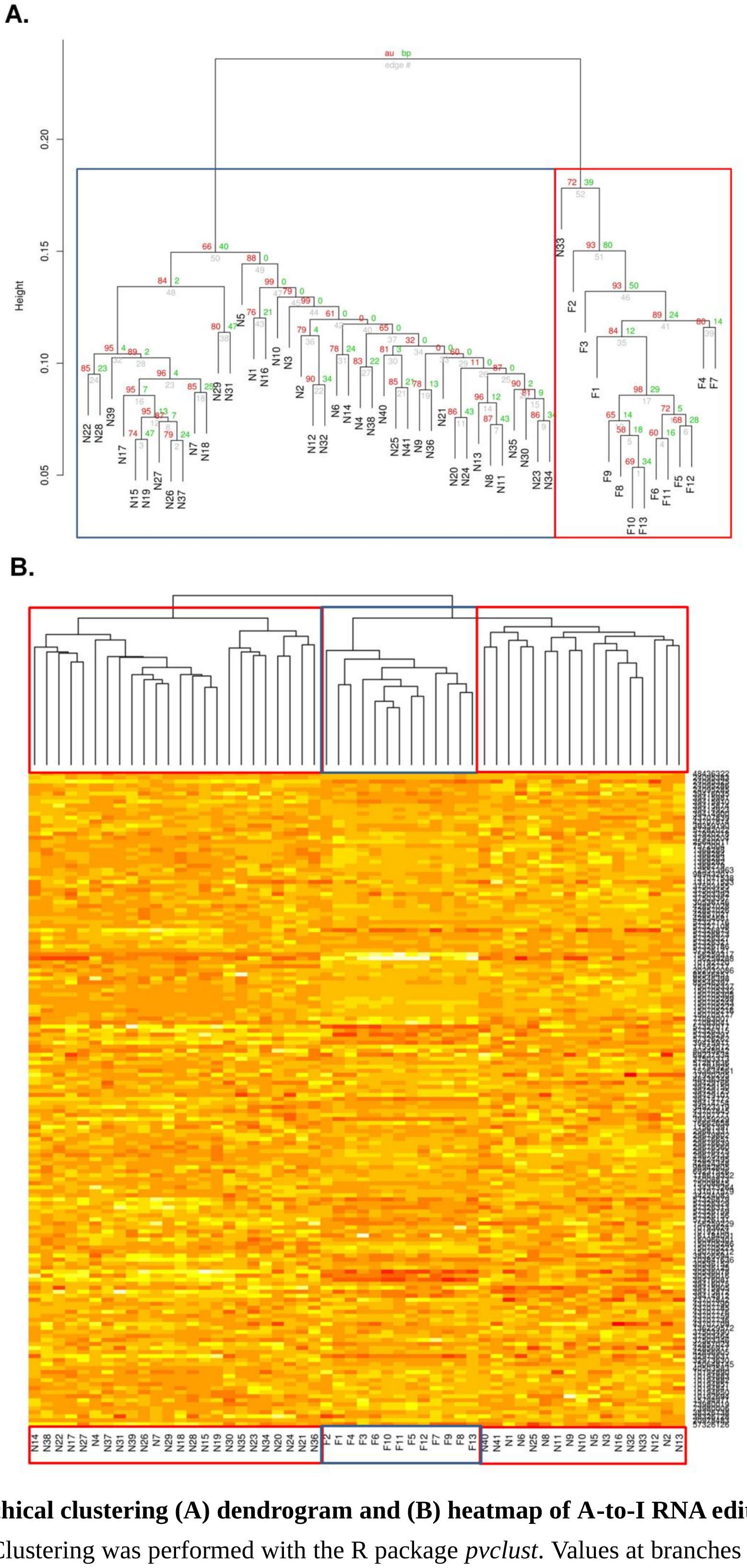

Figure S3. Hierarchical clustering (A) dendrogram and (B) heatmap of A-to-I RNA editing among FL and Normal samples. Clustering was performed with the R package *pvclust*. Values at branches represent multiscale bootstrapping calculated approximately unbiased (AU) *p*-values (red) and bootstrap *p*-values (green). Cluster labels indicating group membership (the real status of the samples: Fi, i=1,2,...13; Nj, j=1,2,...41) are shown below the branches. Membership in the two largest clusters is tracked in the blue (left cluster) and red (right cluster) box, which match the normal (Nj) and NHL (FL, Fi) groups well.

A.

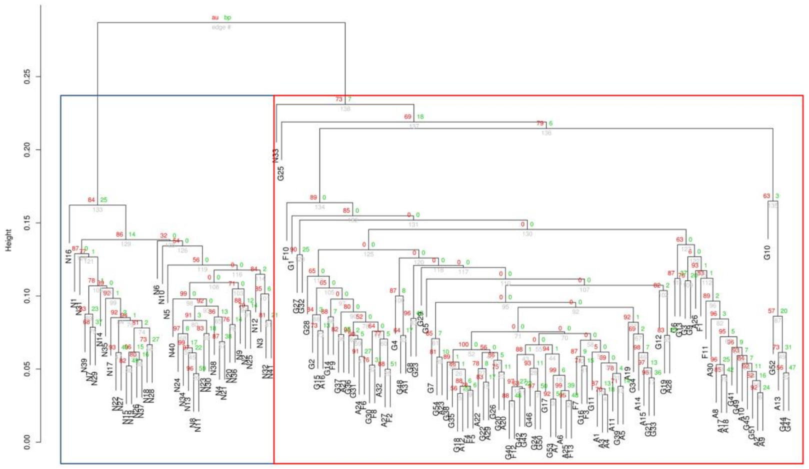

B.

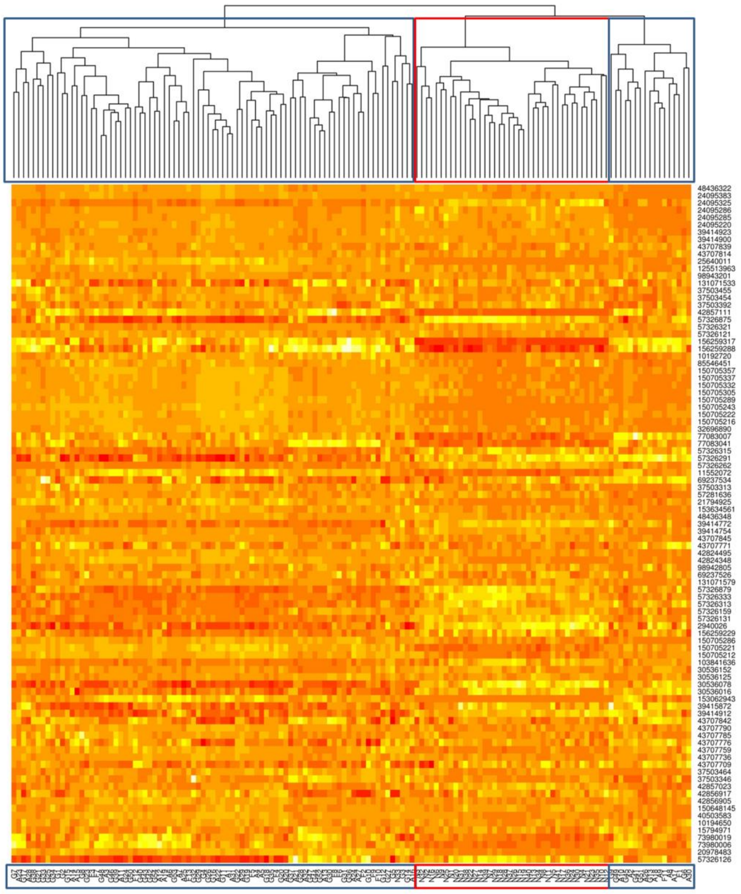

Figure S4. Hierarchical clustering (A) dendrogram and (B) heatmap of A-to-I RNA editing among All NHL and Normal samples. Clustering was performed with the R package *pvclust*. Values at branches represent multiscale bootstrapping calculated approximately unbiased (AU) *p*-values (red) and bootstrap *p*-values (green). Cluster labels indicating group membership (the real status of the samples: Ai, i=1,2,...,32; Gj, j=1,2,...,54; Fk, k=1,2,...13; Nm, m=1,2,...41) are shown below the branches. Membership in the two largest clusters is tracked in the blue (left cluster) and red (right cluster) box, which match the normal (Nm) and NHL (Ai, Gj and Fk) well. However, no clear clusters of NHL subgroups (ABC, GCB and FL) were observed.

Supplementary Tables

Table S1. Detailed lists of all differentially edited sites in all 7 comparisons

(see excel spreadsheet)

Table S2. Detailed number of known editing sites for initial and further filtering steps for all 7 comparison groups

Groups	Number of editing sites after initial filtering	Number of editing sites after further filtering
ABC vs. FL	1789	543
GCB vs. FL	1701	546
GCB vs. ABC	1679	502
NHL vs. Normal	457	398
GCB vs. Normal	538	464
ABC vs. Normal	561	484
FL vs. Normal	579	496

(In total 2576459 for initial filtering)

Table S3. Detailed lists of all differentially edited sites in all 7 comparisons if duplicated reads are not removed in the computational pipeline

(see excel spreadsheet)

Table S4. Clinical status and NCBI accession numbers for all 140 samples in this study

Clinical Status	Accession numbers
GCB (54)	SRX085009, SRX016902, SRX016905, SRX085010, SRX016907, SRX016913, SRX016916, SRX016918, SRX016921, SRX016925, SRX085013, SRX016927, SRX016929, SRX016931, SRX016948, SRX016951, SRX016957, SRX085014, SRX085015, SRX085016, SRX085017, SRX016968, SRX085018, SRX085019, SRX085020, SRX085021, SRX085023, SRX085025, SRX085026, SRX085028, SRX085041, SRX085042, SRX085046, SRX085047, SRX085049, SRX085050, SRX085051, SRX085052, SRX085053, SRX085054, SRX085060, SRX085065, SRX085069, SRX085072, SRX085076, SRX085078, SRX085079, SRX085080, SRX085081, SRX085082, SRX085084, SRX085090, SRX085092, SRX085093
ABC (32)	SRX085011, SRX085012, SRX016923, SRX017248, SRX016935, SRX016938, SRX016946, SRX016953, SRX016955, SRX016962, SRX016964, SRX016966, SRX016970, SRX085022, SRX085027, SRX085040, SRX085045, SRX085048, SRX085055, SRX085056, SRX085057, SRX085058, SRX085059, SRX085067, SRX085074 SRX085077, SRX085085, SRX085086, SRX085087, SRX085089, SRX085091, SRX085094
FL (13)	SRX016940, SRX085029, SRX085030, SRX085031, SRX085032, SRX085033, SRX085034, SRX085035, SRX085036, SRX085037, SRX085038, SRX085043, SRX085044
Normal (41)	SRX014928, SRX014929, SRX014930, SRX014931, SRX014932, SRX014933, SRX014934, SRX014935, SRX014936, SRX014937, SRX014938, SRX014939, SRX014940, SRX014941, SRX014942, SRX014943, SRX014944, SRX014945, SRX014946, SRX014947, SRX014948, SRX014949, SRX014950, SRX014951, SRX014952, SRX014953, SRX014954, SRX014955, SRX014956, SRX014957, SRX014958, SRX014959, SRX014960, SRX014961, SRX014962, SRX014963, SRX014964, SRX014965, SRX014966, SRX014967, SRX014968

Table S5. Lists of all editing sites considered in the analysis including the genes they are associated with

(see excel spreadsheet)
